# The Periplasmic Enzyme, AnsB, of *Shigella flexneri* Modulates Bacterial Adherence to Host Epithelial Cells

**DOI:** 10.1371/journal.pone.0094954

**Published:** 2014-04-24

**Authors:** Divya T. George, Ulrike Mathesius, Carolyn A. Behm, Naresh K. Verma

**Affiliations:** 1 Division of Biomedical Science and Biochemistry, Research School of Biology, The Australian National University, Canberra, Australia; 2 Division of Plant Science, Research School of Biology, The Australian National University, Canberra, Australia; Arizona State University, United States of America

## Abstract

*S. flexneri* strains, most frequently linked with endemic outbreaks of shigellosis, invade the colonic and rectal epithelium of their host and cause severe tissue damage. Here we have attempted to elucidate the contribution of the periplasmic enzyme, L-asparaginase (AnsB) to the pathogenesis of *S. flexneri*. Using a reverse genetic approach we found that *ansB* mutants showed reduced adherence to epithelial cells *in vitro* and attenuation in two *in vivo* models of shigellosis, the *Caenorhabditis elegans* and the murine pulmonary model. To investigate how AnsB affects bacterial adherence, we compared the proteomes of the *ansB* mutant with its wild type parental strain using two dimensional differential in-gel electrophoresis and identified the outer membrane protein, OmpA as up-regulated in *ansB* mutant cells. Bacterial OmpA, is a prominent outer membrane protein whose activity has been found to be required for bacterial pathogenesis. Overexpression of OmpA in wild type *S. flexneri* serotype 3b resulted in decreasing the adherence of this virulent strain, suggesting that the up-regulation of OmpA in *ansB* mutants contributes to the reduced adherence of this mutant strain. The data presented here is the first report that links the metabolic enzyme AnsB to *S. flexneri* pathogenesis.

## Introduction

Shigellosis, more commonly known as bacillary dysentery, is a diarrhoeal infirmity ensuing from an acute inflammatory reaction in the gastrointestinal (GI) tract caused by the enteric bacterium *Shigella*. There are four recognized species of the genus *Shigella*, namely *S. flexneri*, *S. dysenteriae*, *S. boydii*, and *S. sonnei* [1]. Of the four species, *S. flexneri* strains are most frequently linked with endemic outbreaks of shigellosis in the developing world. *S. flexneri* invades the colonic and rectal epithelium of its host and causes severe tissue damage, which manifests in a spectrum of clinical symptoms ranging from watery diarrhea to severe dysentery characterized by fever, abdominal cramping and bloody mucoid stool [2]. Most cases of shigellosis are self-limiting, however in certain individuals, especially malnourished infants, untreated disease can result in several complications which can be fatal [3].

Although a lot has been unraveled about the various steps involved in the pathogenesis of *S. flexneri*, a complete picture of the bacterial genes that are expressed exclusively during infection of host cells has not been elucidated. A clear understanding of bacterial genes expressed at various host-pathogen interfaces can pave the way to the identification of potential attenuation targets and vaccine candidate antigens. In an attempt to identify immunogenic proteins expressed during infection, Jennison and colleagues [4] used 2-dimensional gel electrophoresis (2-DE) followed by immunoblotting with sera from convalescent shigellosis patients, to screen the soluble and membrane protein profiles of *S. flexneri* 2457T. In their study, the chromosomally encoded, metabolic, periplasmic enzyme, L-asparaginase (AnsB) was identified to be immunogenic. This protein is of interest as AnsB has been found to be antigenic in a number of other enteric bacterial species [5]–[8], and has not been characterized in *S. flexneri*, thereby making it a potential attenuation target for vaccine development.

The *E. coli* L-asparaginase (AnsB) protein, which shares 99.1% amino acid identity with *S. flexneri* AnsB, is an enzyme that converts asparagine to aspartate and ammonia. AnsB has high affinity for L-asparagine and the expression of *ansB* is induced by anaerobiosis and positively regulated by the cyclic AMP receptor protein (CRP) and the product of the *fnr* gene [9].

In this study, a reverse genetic approach was used to characterize AnsB in *S. flexneri* and determine if this enzyme is required for bacterial pathogenesis. Recombineering, a lambda red-mediated PCR-based approach [10], was used to knockout the *ansB* gene from a virulent, wild type serotype 3b strain of *S. flexneri*. The effect of *ansB* mutation on bacterial physiology and virulence was analyzed using *in vitro* growth studies, cell culture assays and two *in vivo* models of shigellosis, namely the murine pulmonary model and the *C. elegans* infection model, a new potential animal model of shigellosis [11]. The results of this study indicate that the activity of AnsB is required during the initial stages of bacterial pathogenesis, as *in vitro* bacterial adherence assays clearly showed that *ansB* mutants had decreased adherence to host cells. To gain further insight into how AnsB affects bacterial adherence, we screened the proteomes of the *ansB* mutant and wild type *S. flexneri* strains and found that mutant cells up-regulate the expression of the prominent outer membrane protein, OmpA. These findings highlight the importance of AnsB in the pathogenesis of *S. flexneri*, paving the way to designing new therapeutic or vaccine strategies.

## Materials and Methods

All mice experiments were performed under the protocol approved by the Australian National University Experimentation Ethics Committee.

### Bacterial strains and growth conditions

All *S. flexneri* strains used were derivatives of the highly virulent *S. flexneri* 3b strain, SFL1520, obtained from the International Centre for Diarrhoeal Diseases Research Bangladesh (ICDDRB). Bacterial cultures were grown routinely in Luria Bertani (LB) broth or on LB agar plates. Where appropriate, antibiotic selection was with ampicillin (100 µg/mL) and/or kanamycin (50 µg/mL). For growth studies under nutrient stress, Minimal Essential media (MM) was used. *E. coli* strains were grown and maintained at 37°C, while *S. flexneri* cultures were grown at 30°C to maintain the virulence plasmid.

### Recombinant DNA methods

Restriction endonucleases (New England Biolabs), alkaline phosphatase (Fermentas) and T4 DNA ligase (Promega) were used in accordance with the manufacturer's specifications. Plasmids were maintained in the *E. coli* strain JM109 [12] and isolated using the alkaline lysis method [13] or the QIAprep Spin MiniPrep kit (Qiagen). All primers were synthesized by Sigma-Aldrich. All sequencing was performed at the Biomolecular Resource Facility, Australian National University. PCR was carried out using the high fidelity *PfuUltraII* polymerase (Stratagene) for cloning and sequencing or *iTaq* (Scientifix) for screening.

### Construction of mutant strains

Recombineering was used to generate knockouts in SFL1520. A linear knockout template consisting of the *kanamycin* resistance gene flanked by ∼1000 bp regions showing homology to the upstream and downstream regions of each of the target genes was generated using PCR. Electrocompetent cells of the wild-type SFL1520, transformed with either of the helper plasmids pKD46 or pKM208 [10], were prepared. Freshly prepared electrocompetent cells were transformed with 1–2 µg of each purified knockout template. Transformed cells were recovered in Super Optimal Broth with Catabolite repression (SOC) [14] for 4 hours to allow for homologous recombination. Recovered cells were plated on LB agar containing 50 µg/mL kanamycin and incubated at 30°C for up to 3 days. Resulting colonies were routinely restreaked onto fresh LB agar plates containing 50 µg/mL kanamycin to eliminate false positives. Colonies were then screened for successful gene disruption by colony PCR and sequencing, after which the presence of the virulence plasmid in both mutant strains was confirmed using PCR to amplify two virulence plasmid indicator genes, *apyI* and *virG*. These genes were chosen as they lie on opposite sides of the 220 kb virulence plasmid.

### Growth studies

Overnight cultures of strains were diluted 1∶20 in LB as well as MM and sampled at 30 minute intervals. Growth studies were carried out at 30°C and 37°C under aerobic conditions.

### SDS PAGE and Western Blot

The secretion of type 3 effector proteins was induced using congo red (final concentration 10 mM) for 30 minutes. Bacterial cells were harvested by centrifugation and the supernatants were passed through 0.45 µm filters to eliminate cellular debris. Secretory proteins were precipitated using 25% trichloroacetic acid (TCA) in acetone. Protein precipitates were washed twice using acetone and pellets were allowed to dry completely, after which they were resuspended in solubilization buffer (9 M urea, 4% CHAPS, 1% DTT, 1% ampholytes, 35 mM tris base). Protein concentration was estimated using the BCA kit (Pierce) according to the manufacturer's instructions and 20 µg of proteins in the supernatant samples were loaded onto 12% (v/v) SDS-PAGE gels for Western blots. The gels were stained with Coomassie Brilliant blue R250 to ensure equal loading of samples. Once samples were equalized the separated proteins were transferred onto Hybond-P PVDF membranes (Millipore) and blocked in 5% skimmed milk in Phosphate Buffered Saline (PBS) at 4°C overnight. Following blocking, the membranes were washed thrice in PBS containing 0.05% (v/v) Tween-20 (PBST) (Sigma). Membranes were then incubated with the primary antibodies (Anti-IpaB and Anti-IpaD antisera generated in-house in mice) for 2 hours. Unbound antibodies were washed off using three washes with PBST as outlined above, following which the membranes were incubated with the secondary antibodies (Anti-mouse IgG; Sigma) for 1 hour. Membranes were washed thrice as done previously. The binding of antibodies was then detected by chemiluminescence using SuperSignal West Pico Chemiluminescent Substrate (Pierce) as described by the manufacturer. Chemiluminescence was then detected using the Fisher Biotec chemiluminescence system.

### Reverse transcription polymerase chain reaction (RT-PCR) and Quantitative Real-time Reverse transcription polymerase chain reaction (qRT-PCR)

For both RT-PCR and qRT-PCR, RNA was isolated using Trizol reagent (Invitorgen) according to the manufacturer's instructions. Briefly, overnight cultures of *S. flexneri* strains, (SFL1520, SFL2283 or SFL2443) were diluted 1∶50 and grown to log phase at 37°C. 1×10^9^ CFU were harvested and treated with 1 mL Trizol reagent followed by incubation at room temperature for 5 minutes. Samples were treated with 200 µl chloroform to separate RNA from DNA and proteins. RNA in the aqueous phase was washed with chloroform and precipitated using 2 volumes of isopropanol. RNA pellets were collected by centrifugation and washed using 1 mL freshly prepared 75% ethanol. RNA pellets were air-dried after ethanol wash and suspended in 50 µl of nuclease-free water. The isolated RNA was cleaned up using the Qiagen RNeasy Kit; this step was performed to remove any residual DNA and organic salts. Isolated RNA was treated with Turbo DNase (Ambion) to minimise any contaminating genomic DNA. cDNA was made from 200 ng of RNA using Superscript II (Invitrogen) according to the manufacturer's instructions. qRT-PCR was performed on the cDNA samples using the power SYBR Green RT-PCR kit (Applied Biosystems) according to the manufacturer's instructions except that primers were used at a final concentration of 0.4 µM and the final reaction volume was reduced to 10 µl. Expression of *hisG* was used as a control to normalize the expression of all genes studied. All Real-Time RT-PCRs were performed in triplicate with a no reverse transcriptase (NRT) and a no template control (NTC) set up for each run. Reactions were run in a Rotor-Gene Q Real-Time cycler (Qiagen).

### Measurement of asparaginase activity

Asparaginase activity of SFL1520 and SFL2283 was measured using the ammonia assay kit (Sigma) with log phase culture supernatants, according to the manufacturer's instructions. Bacterial cells expressing L-asparaginase would break down asparagine with the release of ammonia, thus increased ammonia levels correlate with increased hydrolysis of asparagine. Briefly, fresh log phase cells were harvested and washed once in PBS, and 3×10^8^ colony forming units (CFU) of bacteria was resuspended in 1 ml PBS with 5 mM asparagine. Aliquots were taken after 30, 60, 90 and 180 minutes of incubation at 37°C and centrifuged to collect bacterial cells. The ammonia concentration in the cell-free supernatants was measured using the ammonia assay kit, according to the manufacturer's guidelines.

### *In vitro* adherence assay

BHK cells in 6-well plates were infected with 4×10^8^ CFU of each bacterial strain. Bacterial cells were allowed to infect the monolayer for 90 minutes at 37°C, 5% CO_2_. Unbound bacterial cells were washed off the BHK monolayer using PBS, after which the mammalian cells were lysed using 0.05% (v/v) Triton-X in PBS. Appropriate dilutions of the cell lysates were plated on LB agar plates carrying the appropriate antibiotics to determine the number of adherent bacterial cells.

### Coverslip adherence assay

The coverslip adherence assay was performed based on the protocol developed by Cravioto *et al*, [15]. Briefly, overnight cultures of all *S. flexneri* strains grown at 30°C were diluted and grown to log phase at 37°C (OD_600_ = 0.6–0.8). 4×10^8^ CFU of each strain was used to infect a confluent layer of BHK cells grown on coverslips placed within wells of 6-well culture plates. The plates were incubated for 90 minutes at 37°C to allow for infection. The coverslips were then washed thrice in PBS to remove any unbound bacterial cells. Samples were fixed using fresh 70% (v/v) methanol and stained for 30 minutes using 10% (v/v) Giemsa stain. Excess stain was washed off. Coverslips were mounted on glass slides and examined using an oil immersion lens.

### *C. elegans* strains and growth conditions

The *C. elegans* wild type N2 strain [16] was used in this study. Nematodes were maintained at 22°C on modified nematode growth medium (NGM) agar medium seeded with *E. coli* OP50 [16]. A synchronous population of *C. elegans* was established by isolating eggs from adult worms through bleaching [17]. After hatching, L1 worms were transferred to NGM agar plates seeded with *E. coli* OP50 and allowed to grow at 22°C until they reached the L4 stage.

### *C. elegans* bacterial accumulation

All bacterial strains used for the accumulation assay were grown overnight at 37°C on NG agar medium to stimulate expression of virulence plasmid-encoded genes [11]. Plates were cooled to room temperature before they were inoculated with 50–100 nematodes (L4s). The worms were allowed incubated at 22°C for 24 hours after which 10 worms were picked and washed thoroughly using sterile S-basal with 1 mM sodium azide. Worms were then treated with 200 mg/mL gentamycin for 3 hours to remove surface-bound bacterial cells, after which they were washed thoroughly to remove any residual antibiotic. Washed worms were suspended in S-basal +0.1% Triton-X and lysed by mechanical disruption using glass beads as described by [18]. Appropriate dilutions of the lysates were plated onto LB agar with the appropriate antibiotics, to obtain gut bacterial counts. In order to visualize bacterial accumulation within nematode guts, worms were fed *S. flexneri* strains tagged with GFP^+^. Following 24 hours of infection, bacterial fluorescence was observed using the EVOS digital inverted microscope (AMG) and quantified using the Infinite M1000 Pro (Tecan).

### *C. elegans* survival assays

Overnight cultures of *S. flexneri* strains maintained at 30°C were diluted 1: 50 in LB media and grown to log phase at 37°C. A synchronized population of L4 stage *C. elegans* worms was treated with 200 µg/mL gentamycin for 3 hours, after which they were washed thoroughly with S-basal to remove any residual antibiotic. Approximately 20 washed L4s were transferred into each well of a 24-well plate containing 100 µL of the appropriate log-phase bacterial culture to be tested. The volume of solution in each well was adjusted to 500 µL with S-basal and plates were incubated at 22°C. The number of live worms in each well was scored every 12 hours and the percentage survival was calculated. Worms that showed no pharyngeal pumping and remained immobile on tapping the plate were considered dead. A minimum of three replicates for each test strain was set up per trial.

### Mouse pulmonary model of shigellosis

6–8 week old female Balb/c mice weighing approximately 20–25 g (Animal Resource Centre, WA) were inoculated intranasally with a sub-lethal dose (2×10^7^ CFU/10 µL) of either the pathogenic wild type SFL1520 strain or SFL2283 (Δ*ansB*). The mice were lightly anesthetized with isoflurane prior to inoculation. 20 mice were euthanized 24 hours post infection and their lungs were extracted and homogenized in PBS with 0.05% Triton-X. Appropriate dilutions of the homogenates were plated on LB agar with antibiotics, where appropriate, to obtain the live counts of intracellular bacteria per lung.

### Isolation of total bacterial protein for two-dimensional gel electrophoresis

Overnight cultures of SFL1520 (wild type parent) and SFL2283 (Δ*ansB*) grown in LB at 30°C were diluted 1/100 and grown to log phase at 37°C. Bacterial cells were collected and washed thrice using cell wash solution (10 mM Tris pH 8.0, 5 mM magnesium acetate). Bacterial pellets were resuspended in 500 µl of cell lysis buffer containing 7 M urea, 2 M thiourea, 30 mM tris-base, 4%(w/v) CHAPS and Complete Protease Inhibitor (Roche) and incubated on ice for 10 minutes. Subsequently, the suspensions were sonicated 10 times for 15 s with 30 s intervals, on ice. Cellular debris was sedimented by centrifugation. Supernatants were treated with 10 volumes of ice-cold acetone and incubated at −20°C overnight to precipitate proteins. The protein pellets were washed thrice using ice-cold acetone, following which they were dried. Acetone-free proteins were solubilized in 200 µl of cell lysis buffer and pH was adjusted to 8.5. For 2-dimensional (2-D) gel electrophoresis, total protein from wild type and *ansB* mutant strains were isolated in triplicate over three independent experiments. Protein concentration was determined using the BCA kit (Pierce) and 500 µg of each protein sample was labeled with fluorescent dyes Cy3 or Cy5 (GE Healthcare); an internal standard, consisting of a mixture of 250 µg of each sample, was labeled with Cy2. Samples were labeled with the CyDye DIGE Fluors (minimal dyes) for Ettan DIGE kit (GE Healthcare), according to the manufacturer's instructions, with minor modifications as described by Mathesius *et al*, 2001. Briefly, 500 µg of each protein sample was labeled with 400 pmol amine reactive cyanine freshly dissolved in anhydrous dimethyl formamide. CyDye labeling was carried out on ice and in the dark for 30 minutes. Each reaction was terminated using 10 nmol lysine to eliminate any unbound dye. Labeled samples were treated with DTT (20 mg/ml) and Bio-ampholytes (50 µl/ml). To identify differences in the proteomes of SFL1520 and SFL2283, 500 µg of total protein from the wild type parent (SFL1520) labeled with Cy3 or Cy5 was combined with 500 µg of oppositely labeled proteins from the Δ*ansB* mutant strain. Each wild type-mutant pair was then mixed with 500 µg of the combined protein preparations of all protein samples that were labeled with Cy2 as an internal control.

### 2-D electrophoresis

2-D electrophoresis was performed in darkness to maintain the stability of the Cy dyes. Immobiline pH 3–10 NL Drystrips (24 cm, GE Healthcare) were used for the first dimension isoelectric focusing (IEF). The strips were rehydrated overnight in rehydration solution containing, 8 M urea, 0.5% (w/v) CHAPS, 0.2% (w/v) DTT, 0.52% (w/v) bio-ampholytes and 0.6% (w/v) bromophenol blue. Wild type-mutant pairs with internal controls were loaded onto rehydrated Immobiline strips and IEF was carried out in a Multiphor II electrophoresis system (GE Healthcare) at 20°C for a total of 35,000 volt hours [19].

For separation of proteins across the second dimension, self-cast 12.5% SDS-PAGE gels were cast using the EttanDALTsix system (GE Healthcare). The gels were cast using low fluorescence glass plates, which are compatible with visualizing the CyDyes. Focused first dimension strips were equilibrated as described by [19] and placed on the second dimension gels. SDS-PAGE was carried out at 10°C in SDS running buffer (25 mM Tris, pH 8.0, 192 mM glycine and 0.1% SDS) at 600 V, 10 mA, and 2.5 W per gel for the first hour; 600 V, 40 mA, and 13 W per gel until the bromophenol blue front reached the bottom of the gel.

### Gel imaging and image analysis

After the second dimension electrophoresis, DIGE-labeled proteins were visualized using a Typhoon Trio laser scanner (GE Healthcare). Gels were scanned with the specific excitation wavelengths of Cy3 (532-nm laser and a 580-nm band pass 30 emission filter), Cy5 (633-nm laser and a 670-nm band pass 30 emission filter) and Cy2 (488-nm laser and a 580-nm band pass 40 emission filter). Spot detection and analysis was carried out using DeCyder Version 5 (GE Healthcare) software followed by careful manual confirmation and rematching of matching errors. Statistics and identification of differentially expressed spots were carried out in the DeCyder DIA and BVA modules (one-way ANOVA).

### In-gel trypsin digestion of protein spots and mass spectrometry

After analysis of gel images, identified spots of interest were excised from the 2D-DIGE gels using an Ettan spot picker (GE Healthcare). The spot picker was calibrated and tested several times prior to spot excision. In-gel trypsin digest was carried out as described by Mathesius *et al*, 2001 with a few modifications. Briefly, excised protein spots were washed four times in acetonitrile: 50 mM ammonium bicarbonate (50∶50, v/v). Spots were then dried in 100% acetonitrile for 30 minutes following which they were air-dried to eliminate all acetonitrile. Gel pieces were then rehydrated with a trypsin solution (20 units; Promega) and incubated for 2 hours at 4°C followed by overnight incubation at 37°C. Peptides were then extracted from the gel pieces using an extraction buffer consisting of acetonitrile: water: trifluroacetuic acid (TFA) (50%∶50%∶1%, v/v) followed by gentle sonication in a sonic water bath for 40 minutes. This extraction step was performed twice using a reduced volume of extraction buffer and 20 minutes sonication. Peptides were collected and dried completely to remove all traces of TFA. Dried peptides were resuspended in 20 µl of acetonitrile∶water∶formic acid (10%∶89.9%∶0.1%, v/v) of which 6 µl were loaded onto the mass spectrometer. The peptides were identified at the Mass Spectrometry Facility, The Australian National University, on an Agilent 6530 Q-TOF LC/MS (Santa Clara, CA, USA) with a ChipCube ion source interface (Agilent Technologies, Inc., Palo Alto, CA) containing a liquid chromatographic chip (ProtID-Chip-150(II), separation: 150 mm×75 µm, enrichment: 4 mm 40 nL, packed with 5 µm Zorbax 300SB-C18 particles) The LC separation system included a binary capillary pump operated at a flow rate of 4 µL/min, used for loading the samples, and a nanoflow gradient pump using a linear gradient from 8 to 38% mobile phase B in 47 min at a flow rate of 300 nL/min. The column was then washed with 90% mobile phase B for 5 min. Mobile phase A 0.1% formic acid and mobile phase B was 90% acetonitrile/water containing 0.1% formic acid.

The spray from the chip was subjected to positive polarity electrospray ionisation (ESI) using the following settings: gas flow rate 4 L/min, gas temperature 300°C, capillary voltage 1900 V, fragmentor 175 V, skimmer 65 V and octopole RF peak 750 V. The instrument was run in extended dynamic range mode with data dependent acquisition switching between MS (*m/z* 100–1700 at 3 spectra/s) and MS/MS (*m/z* 50–1700 at 3 spectra/s), measuring the collision induced dissociation (CID) fragment spectra of the three most intense precursor ions with charge states 2, 3 and ≥3 with a 15 s dynamic exclusion time, within a cycle time of 1.4 s. The collision energy was automatically set by the Agilent MassHunter Acquisition software (slope 3, offset 2). The *m*/*z* values of all ions present in the mass spectra were corrected against two reference ions (purine, [MH]^+^ *m*/*z* 112.985587 and 1H, 1H, 3H tetra(fluoropropoxy)phosphazine, [MH]^+^ *m/z* 922.0097). Data was acquired and analysed with Agilent Technologies MassHunter software (version B.4.0).

Proteins were identified through Peptide Mass Fingerprinting using MASCOT (Matrix Science). One missed cleavage per peptide was allowed and a mass tolerance between 0.3 and 0.1 Da was used in all searches. Carbamidomethylation was set as a fixed modification and oxidation (M) as a variable. We searched the Ludwig database under taxonomy *Shigella flexneri* on the MASCOT database at the Australian Proteomic Computation Facility (APCF).

## Results

### Insertional inactivation of *ansB* in the wild type *S. flexneri* serotype 3b strain

The *ansB* gene was successfully disrupted in wild type *S. flexneri* serotype 3b (SFL1520) using recombineering to produce *ΔansB* (SFL2283). Excessive handling and manipulation of *S. flexneri* strains often results in the loss of the virulence plasmid, therefore the presence of the virulence plasmid in *S. flexneri* serotype 3b (SFL1520) and *ΔansB* (SFL2283) was confirmed by PCR before every virulence assay performed in this study as described in the methods.

*S. flexneri* virulence-plasmid encoded invasion protein antigens (Ipa) are important components of the type three secretion needle complex, required for virulence. In order to determine whether *ΔansB* (SFL2283) efficiently expressed and secreted virulence-plasmid encoded proteins, we investigated the expression and secretory levels of IpaB and IpaD, two key components of the type three secretory apparatus, using western immunoblots. Western immunoblots showed no differences in the expression and secretion of IpaB and IpaD in *ΔansB* (SFL2283) and the wild type serotype 3b strains (SFL1520) (Figure S1), thus suggesting that expression and secretion of key virulence plasmid-encoded proteins remain unaffected by the *ansB* mutation.

**Figure 1:**
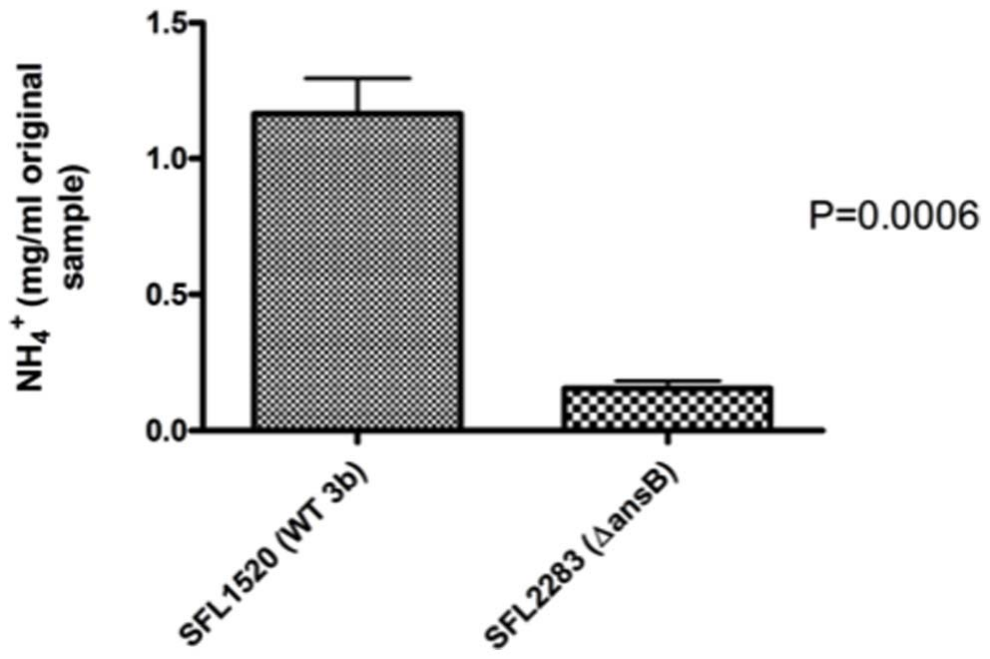
AnsB in *S. flexneri* hydrolyses asparagine. *S. flexneri* parental strain SFL1520 or isogenic mutant *ΔansB* (SFL2283) at 3×10^8^ colony forming units (CFU) per ml were incubated in phosphate buffered saline (PBS) with 5 mM asparagine. Ammonia production was measured after 60 minutes incubation at 37°C. Hydrolysis of asparagine was measured by quantifying ammonia production. There is a significant drop in the levels of released ammonia by *ansB* mutant cells (*p* = 0.0006, unpaired t-test). Error bars represent the standard error of means obtained from three independent experimental measurements.

Prior to carrying out any physiological studies, we confirmed that the *ansB* gene was expressed in wild type serotype 3b (SFL1520) when grown *in vitro* under nutrient rich and starved conditions, using reverse transcriptase PCR (RT-PCR) (data not shown). AnsB is an identified L-asparaginase in *E. coli*; to determine if this function was conserved in *S. flexneri*, we used an ammonia assay to measure levels of ammonia released when bacterial cells were treated with asparagine. Ammonia assays showed that wild type *S. flexneri* serotype 3b (carrying intact *ansB*) released significantly more ammonia than the Δ*ansB* strain (Figure 1). These results suggest that AnsB in *S. flexneri* functions as an L-asparaginase.

### The activity of AnsB is not essential for bacterial growth *in vitro*

Once we established that the *ansB* gene was expressed and functional in *S. flexneri* serotype 3b when grown *in vitro*, we carried out *in vitro* growth studies to determine whether the metabolic activity of AnsB was required for bacterial growth. No mutation-related growth retardation was observed when strains were grown under nutrient rich (LB media) conditions at both 37°C and 30°C (Figure S2.A). To determine if *ansB* expression was required for growth under stress conditions, growth studies were carried out under reduced nutrient conditions using minimal essential salts media (MM) (Figure S2.B). No significant (*p*>0.05, unpaired t-test) growth retardation was exhibited by the *ansB* mutant strain, suggesting that, although this metabolic gene is expressed, its activity is not vital for bacterial growth *in vitro*.

### AnsB activity is required for *S. flexneri* adherence to baby hamster kidney (BHK) cells

**Figure 2:**
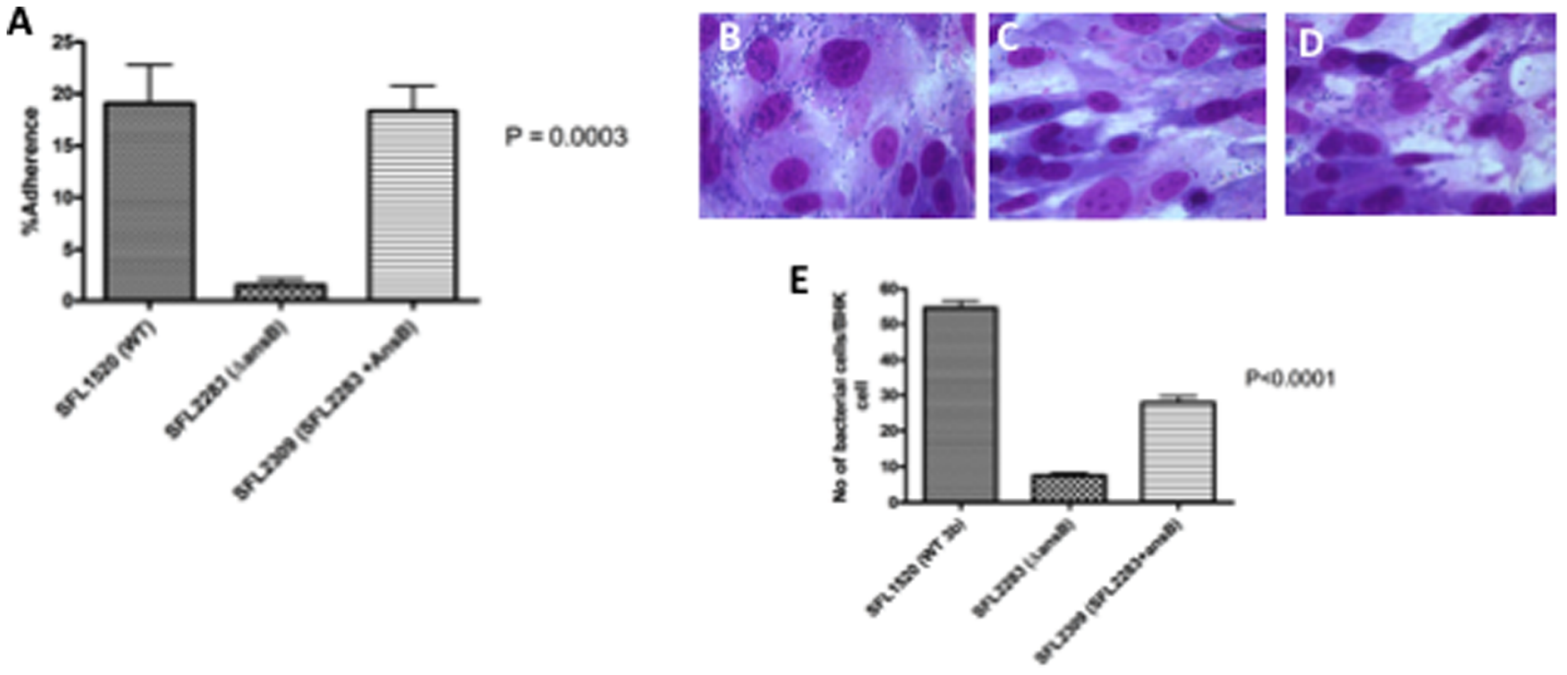
AnsB activity required for *S. flexneri* adherence to baby hamster kidney (BHK) cells. Results of *in vitro* adherence assays for wild type *S. flexneri* serotype 3b (SFL1520), Δ*ansB* (SFL2283) and complemented strain SFL2309 (SFL2283 + *ansB*). **A:** Bacterial adherence to baby hamster kidney cells plotted as percentage adherence, a ratio of the number of adherent and intracellular bacteria to the total number of bacterial cells in the infecting inoculum (y-axis). SFL2283 cells are significantly less adherent that SFL1520 and SFL2309 (*p* = 0.0003, One Way ANOVA). Results are the means of three independent blind repeats with standard errors. **B–D:** Results of coverslip adherence assay performed to compare the adherence of *S. flexneri* 3b wild type strain (SFL1520-**B**), Δ*ansB* (SFL2283-**C**) and complement of Δ*ansB* (SFL2309-**D**). **E:** A graph depicting the number of adherent bacteria counted per BHK cell under 1000× magnification (y-axis). The adherence of SFL2283 is significantly lower than SFL1520 and SFL2309 (*p*<0.0001, One Way ANOVA). Results are the mean of 10 cells across 10 fields with standard errors, counted at random in a blind experiment.

*in vitro* adherence assays were performed to investigate whether the activity of AnsB contributed to bacterial adherence to epithelial cells. The Δ*ansB* (SFL2283) strain showed a significant drop in the number of adherent bacteria when compared with the wild type parental strain (SFL1520) (Figure 2.A). This decrease in adherence was restored upon complementation of the *ansB* mutation in SFL2309 (SFL2283 + plasmid expressing *ansB*).

In order to visualize the differences in adherence patterns of the mutant and wild type strains, a coverslip adherence assay was performed. Giemsa staining of infected BHK monolayers also showed a decrease in the number of adherent bacteria in monolayers infected with Δ*ansB* (SFL2283) (Figure 2.C) compared with wild type *S. flexneri* 3b (SFL1520) (Figure 2.B) and the ansB complemented strain SFL2309 (Figure 2.D). The number of adherent bacteria per BHK cell was determined in order to validate the microscopic findings. Δ*ansB* (SFL2283) cells showed a significant drop in the number of adherent bacterial cells when compared with SFL1520 and SFL2309 (Figure 2.E).

### AnsB activity is required for *S. flexneri*-mediated killing and bacterial accumulation in the intestinal lumen of *C. elegans*

**Figure 3:**
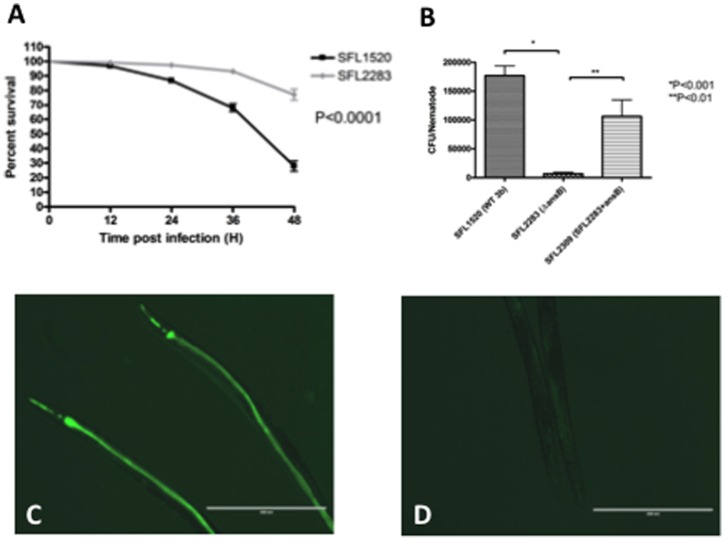
AnsB activity required for *S. flexneri*-mediated killing and bacterial accumulation in *C. elegans*. **A**: *S. flexneri*-mediated *C. elegans* liquid killing assays. 20 synchronized adult nematodes were treated with log phase cultures of *S. flexneri* 3b (SFL1520-black) or Δ*ansB* (SFL2283-grey) strains grown at 37°C to express virulence factors. Infected nematodes were monitored for 48 hours and survival was scored every 12 hours. Worms treated with the Δ*ansB* strain show a significant increase (*p*<0.0001, Logrank test) in survival rates compared to nematodes infected with wild type *S. flexneri* 3b. Error bars represent standard error of mean across at least 3 independent biological repeats. **B**: Bacterial accumulations assays; young adult hermaphrodite nematodes were fed SFL1520, SFL2283 or *ansB* complemented strain SFL2309 (SFL2283 + *ansB*) for 24 hours. 20 nematodes were picked and mechanically disrupted to release internalized bacteria. Diluted lysates were plated on LB agar plates carrying appropriate antibiotics, and colonies were scored in order to quantify *S. flexneri* cells associated with each nematode. There is a significant decrease in the accumulation of SFL2283 compared to SFL1520 and SFL2309 (*p*<0.01, One-way ANOVA). Error bars represent standard error of mean across 3 independent biological repeats. Young adult hermaphrodite nematodes infected as above with GFP^+^- tagged SFL1520 (**C**) and SFL2283 (**D**). Wild type *S. flexneri* 3b accumulates in the nematode gut lumen and Δ*ansB* (SFL2283) strain fails to accumulate in the worm gut lumen. Scale bar = 400 µm.

To determine if the reduced adherence observed *in vitro* in the Δ*ansB* (SFL2283) strain had an effect on bacterial virulence; preliminary *in vivo* studies were carried out in *C. elegans*. Kesika and colleagues have previously reported that virulent *S. flexneri* serotype 2b kills *C. elegans* in liquid culture [18]. We performed liquid killing assays to compare the killing rates of Δ*ansB* (SFL2283) and wild type *S. flexneri* serotype 3b. *C. elegans* fed wild type *S. flexneri* 3b (SFL1520) died much faster (with 50% killing (TD_50_) = 42±1 h) than worms maintained on Δ*ansB* (SFL2283) (<50% killing in 48 h) (Figure 3.A).

Burton E.A. *et al* [11] have demonstrated that virulent *S. flexneri* strains accumulate in the intestinal lumen and kill *C. elegans*, while avirulent *S. flexneri* strains are digested by the nematodes. To determine whether the survival of nematodes fed the *ΔansB* strain correlated with low intraluminal bacterial numbers, *C. elegans* bacterial accumulation assays were performed. Results of bacterial accumulation assays correlate with nematode killing, as the wild type *S. flexneri* 3b strain (SFL1520) accumulated within the gut of the worms, while Δ*ansB* (SFL2283) cells were digested by the nematodes (Figure 3.B). Complementation of the *ansB* mutation restored the intestinal accumulation phenotype of the wild type strain.

In order to visualize bacterial accumulation, nematodes were fed GFP^+^ tagged *S. flexneri* strains. Consistent with the bacterial accumulation assays, worms fed wild type *S. flexneri* 3b (SFL1520) showed accumulation of GFP^+^ tagged bacterial cells within their intestinal lumen (Figure 3.C), while those fed the Δ*ansB* (SFL2283) strain failed to show GFP^+^ accumulation (Figure 3.D). GFP^+^ accumulation assays could not be carried out using the complemented strain as the plasmid expressing GFP^+^ was not compatible with the plasmid used for the complementation of *ansB* mutation.

The enhanced survival of nematodes fed Δ*ansB* (SFL2283) and digestion of wild type *S. flexneri* 3b following mutation of *ansB* suggests that this metabolic enzyme is required for bacterial virulence.

### AnsB activity is required for *S. flexneri* infection in the murine pulmonary model of shigellosis

**Figure 4:**
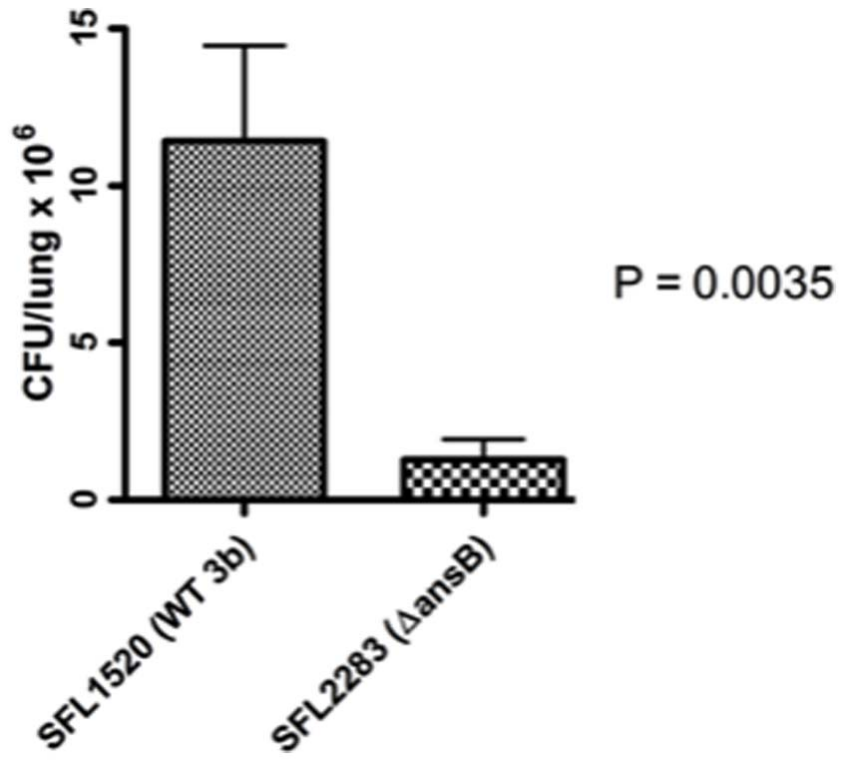
AnsB activity required for *S. flexneri* infection in the murine pulmonary model of shigellosis. Lung counts of mice inoculated intranasally with a sub-lethal dose (2×10^7^ CFU) of *S. flexneri* 3b (SFL1520) and Δ*ansB* (SFL2283). 20 mice per strain were euthanized 24 hours post-inoculation, and their lungs were isolated and homogenized. Dilutions of the homogenates were plated to determine the number of intracellular bacterial cells 24 hours post inoculation. Results represent the mean values of lung counts obtained from 20 mice infected with each strain in a blind experiment with standard errors (error bars). There is a significant decrease in the lung counts of mice infected with the *ansB* mutant strain compared to mice infected with the wild type strain (*p* = 0.0035, unpaired t-test).

Since preliminary *in vivo* studies in *C. elegans* indicated that the *ansB* mutant showed reduced virulence, the virulence of this strain was also assessed using the murine pulmonary model, an established animal model of shigellosis [21]–[23]. Virulent strains of *S. flexneri*, when inoculated intranasally, colonize the lungs of infected mice and cause pneumonia. The lung counts obtained from mice inoculated with the Δ*ansB* strain 24 hours post infection were significantly lower than the wild type strain (*p* = 0.0035, unpaired t tests) (Figure 4), further demonstrating that AnsB activity is required for the virulence of *S. flexneri*.

### 2-Dimensional gel electrophoresis identifies changes in the *S. flexneri* proteome caused by *ansB* mutations

**Figure 5:**
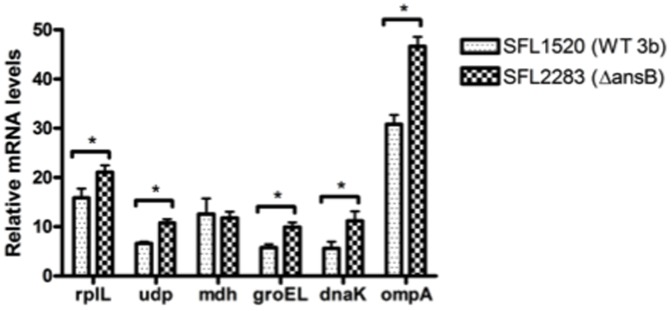
Reverse transcription quantitative PCR (qRT-PCR) analysis to confirm two dimensional differential in-gel electrophoresis (DIGE) analysis. Transcript levels of proteins identified by DIGE analysis, *rplL*, *udp*, *mdh*, *groEL*, *dnaK* and *ompA*, were measured in Δ*ansB* (SFL2283) and wild type *S. flexneri* serotype 3b (SFL1520) and expressed as a ratio of the *ansB* mutation-induced levels and the basal wild type levels. Data represent the means of three independent biological repeats, each replicate measured in triplicate and normalized to the control gene *hisG*. Asterisks indicate statistically significant differences calculated using unpaired Student's t-test (*p*<0.05).

To gain insight into why the *ΔansB* strain shows reduced adherence to epithelial cells, we used two dimensional differential in-gel electrophoresis (DIGE) to compare the proteomes of Δ*ansB* (SFL2283) and wild type *S. flexneri* serotype 3b (SFL1520) strains. Our proteomic studies identified 17 differentially expressed proteins in Δ*ansB* (SFL2283). Using liquid chromatography mass spectrometry (LC-MS) we identified 6 (out of 17) proteins as up-regulated in the Δ*ansB* (SFL2283) strain (RplL, Udp, Mdh, OmpA, GroEL and DnaK) (Table S1). Quantitative real-time reverse transcription polymerase chain reaction (qRT-PCR) was performed to confirm the results obtained in the DIGE analysis (Figure 5). We analysed the transcript levels of proteins identified in the DIGE analysis, in three independent biological replicates, using the bacterial *hisG* gene as a control gene to normalize the expression of all genes tested. Similar to our DIGE analysis, the expression of *rplL*, *udp*, *ompA*, *groEL* and *dnaK* was up-regulated in the Δ*ansB* (SFL2283) strain. However, no significant differences in the transcript levels of *mdh* were observed (Figure 5).

### Overexpression of the outer membrane protein OmpA in wild type *S. flexneri* serotype 3b decreases bacterial adherence to BHK cells and virulence in *C. elegans*

**Figure 6:**
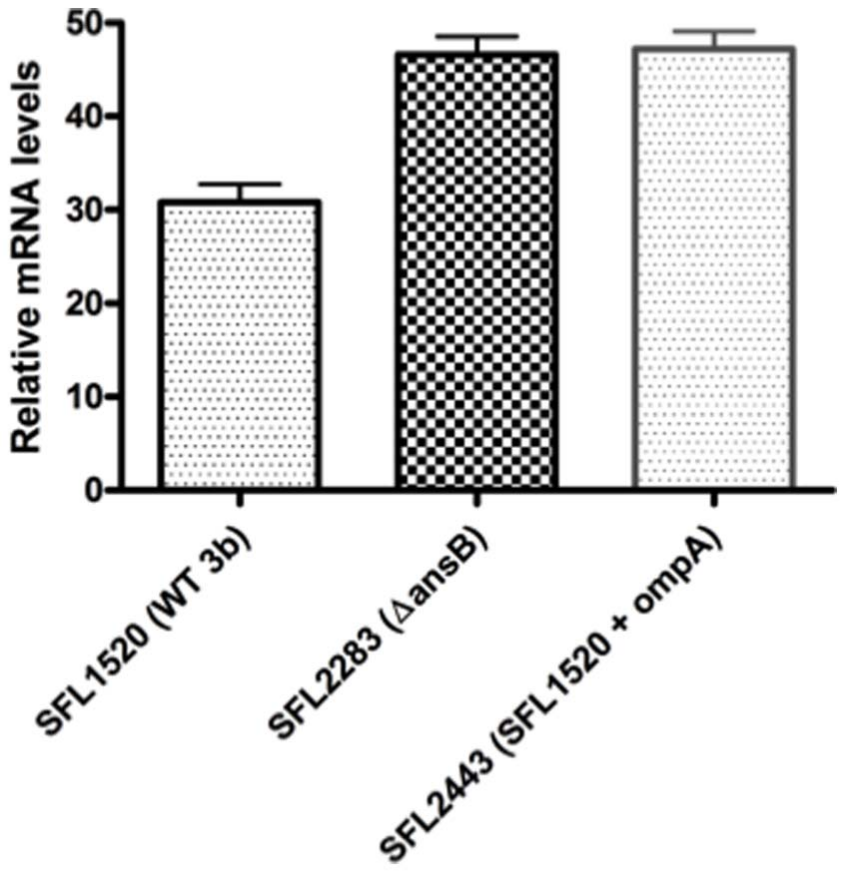
qRT-PCR analysis confirms the up-regulation of *ompA* in SFL2443 (wild type + *ompA*). qRT-PCR was used to compare the mRNA transcript levels of *ompA* in SFL1520 (wild type *S. flexneri* serotype 3b), SFL2283 (Δ*ansB*) and SFL2443 (SFL1520 + a plasmid carrying *ompA* to account for the up-regulation of OmpA seen in the Δ*ansB* mutant). Results represent the means of three biological repeats, each measured in triplicate and normalized to the control gene *hisG*. Error bars indicate the standard errors of the means. OmpA mRNA levels in SFL2443 are significantly higher than SFL1520 (*p*<0.001, unpaired t-test) and similar to the up-regulated levels seen in SFL2283 (*p*>0.05, unpaired t-test).

Since the *ansB* mutant exhibited decreased adherence and up-regulation of OmpA, a prominent outer membrane protein, we decided to determine whether the up-regulation of OmpA in Δ*ansB* was responsible for the observed virulence phenotypes. To investigate whether the up-regulation of OmpA had an effect on bacterial virulence, we overexpressed OmpA in wild type 3b (SFL1520) by introducing a plasmid carrying *ompA* into SFL1520, to generate SFL2443 (SFL1520 + *ompA*). qRT-PCR was used to confirm that there was an increase in *ompA* transcripts in SFL2443 compared with the wild type strain (Figure 6). SFL2443 was then used to perform cell culture adherence assays and *in vivo C. elegans* virulence assays in order to assess whether the decreased adherence shown by the *ansB* mutant strain was a result of the up-regulation of outer membrane protein, OmpA.

**Figure 7:**
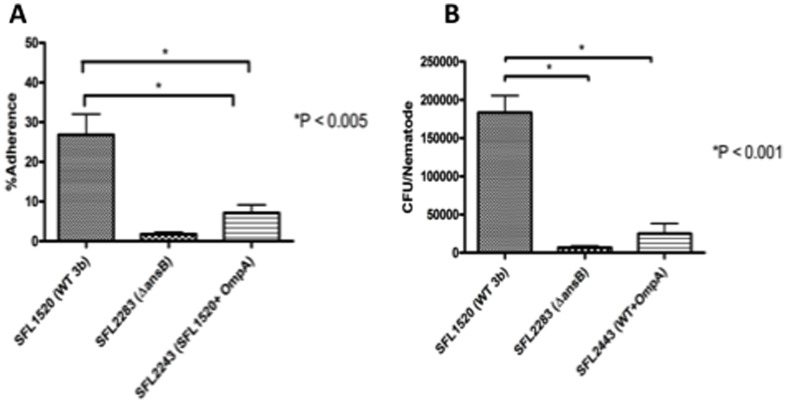
Overexpression of OmpA decreases *S. flexneri* adherence *in vitro* and virulence in *C. elegans*. **A**: Results of *in vitro* adherence assays for SFL2443 (SFL1520 + a plasmid expressing *ompA* to account for the up-regulating of OmpA seen in the Δ*ansB* mutant), wild type *S. flexneri* 3b (SFL1520), and Δ*ansB* (SFL2283) strains plotted as percentage adherence, a ratio of the number of adherent and intracellular bacteria to the total number of bacterial cells in the infecting inoculum (y-axis). The adherence of SFL1520 is significantly decreased as a result of the overexpression of OmpA in, SFL2443 (*p*<0.005, unpaired t-test). Results are the means of three independent blind repeats with standard errors (error bars).**B:** Results of *in vivo C. elegans* bacterial accumulation assays for SFL2443, SFL1520, SFL2283 strains. The accumulation of SFL1520 is significantly higher than SFL2443 (*p*<0.001, unpaired t-test). Results are the means of three independent blind repeats with standard errors (error bars).

Adherence assays performed using SFL2443 (SFL1520 + *ompA*) clearly indicate that the overexpression of OmpA in wild type *S. flexneri* serotype 3b significantly decreased the adherence of wild type cells (Figure 7.A). Similarly, *in vivo* bacterial accumulation assays in *C. elegans* showed that the overexpression of OmpA in wild type 3b, reduced bacterial accumulation in the nematodes to levels similar to *ΔansB* (SFL2283) (Figure 7.B). Results of both the BHK adherence assays and *C. elegans* bacterial accumulation assays suggest that the up-regulation of the outer membrane protein OmpA is responsible for the decreased virulence of the *ΔansB* strain.

### *ansB* regulates the expression of *ompA* in *S. flexneri*

**Figure 8:**
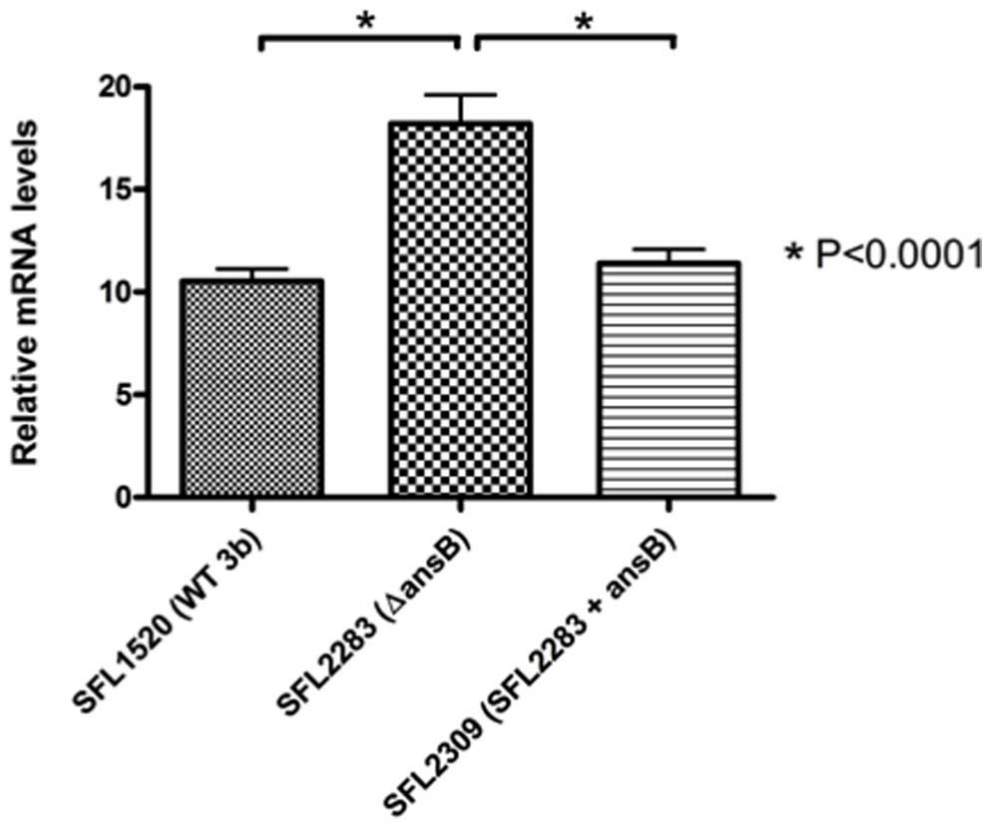
qRT-PCR analysis confirms that the presence of *ansB* regulates the expression of *ompA* in *S. flexneri*. qRT-PCR was used to compare the mRNA transcript levels of *ompA* in SFL1520 (wild type *S. flexneri* serotype 3b), SFL2283 (Δ*ansB*) and SFL2309 (SFL2283 + a plasmid carrying *ansB* to complement the *ansB* mutation). Results represent the means of three biological repeats each measured in triplicate and normalized to the control gene *hisG*. Error bars indicate the standard errors of the means. A significant increase in *ompA* expression is observed in SFL2285 (*p*<0.0001) when compared with SFL1520 and SFL2309. No significant differences were observed in the expression of *ompA* between the wild type parental strain (SFL1520) and the *ansB* complement (SFL2309) (*p*>0.05, unpaired t-test), suggesting that complementation of the *ansB* mutation in SFL2309, restores the expression of this gene to wild type levels.

Since DIGE analysis indicated that OmpA levels were elevated in *ansB* mutant cells and virulence studies showed that the up-regulation of OmpA in wild type cells lead to decreased bacterial adherence, we decided to determine if the presence of AnsB does in fact affect the expression of *ompA*. In order to determine whether the presence of *ansB* had an effect on the expression of *ompA* in *S. flexneri*, qRT-PCR was performed to compare the levels of *ompA* transcripts in the wild type 3b strain, *ansB* mutant and the *ansB* mutant strain complemented with a plasmid copy of *ansB*. Using this approach, we show that on complementation of the *ansB* mutation, the levels of *ompA* transcripts are restored to wild type levels (Figure 8). These results suggest that *ansB* does in fact play a role in the regulation of *ompA* expression.

## Discussion

Here we characterize AnsB, a metabolic enzyme identified to be immunogenic in *S. flexneri* [4] and found that the activity of this enzyme is required for the virulence of *S. flexneri*. Using *in vitro* bacterial adherence assays and two *in vivo* models of shigellosis, we found a marked decrease in the adherence and virulence of the *ansB* mutant strain (SFL2283), clearly suggesting that AnsB is involved in the initial stages of *S. flexneri* pathogenesis.

The L-asparaginase activity of AnsB has previously been found to enhance bacterial colonization of mammalian cells by increased hydrolysis of asparagine in the case of *Campylobacter jejuni* [24]–[26] and *Helicobacter pylori* [6], [27]. However, the role of this enzyme in *S. flexneri* virulence has not been elucidated. The results of ammonia release assays suggest that AnsB in *S. flexneri* functions as an L-asparaginase, as is the case in *E. coli* [9] and *Salmonella enterica* [28]. This reaction features in several metabolic pathways, including nitrogen, cyanoamino acid, alanine and aspartate metabolism [29].

The involvement of L-asparaginase activity in several metabolic pathways suggests that mutating *ansB* could lead to metabolic impairments, which in turn could decrease overall bacterial fitness. However, our *in vitro* growth studies found no differences in the growth rates of mutant and wild type cells, which suggests that although *ansB* is expressed and functional in wild type *S. flexneri* cells grown *in vitro*, its activity is not required for bacterial growth *in vitro* under nutrient rich or nutrient stressed conditions (Figure S2). The results of our growth studies are not surprising as AnsB in *E. coli*, is essential for growth under low oxygen, poor carbon source conditions by providing an alternative electron acceptor. Therefore although this gene is expressed in *S. flexneri* cells grown aerobically, the activity of this enzyme may only be critical under anaerobiosis [30]. Therefore further growth studies need to be performed under anaerobic conditions in order to determine whether *ansB* mutant cells show reduced bacterial fitness.

Successful establishment of bacterial infection requires adherence to host tissue. Members of the family *Enterobacteriaceae* use a plethora of strategies to adhere to host tissues, ranging from the use of pili to the secretion of highly specialized adhesion molecules [31]. The molecular mechanisms used by *S. flexneri* to adhere to host cells are relatively unknown. Previous studies have shown that the bacterial Type III secretory Ipa proteins, especially IpaB, facilitate adherence to mammalian tissue [32]–[34]. In this study, western immunoblots confirmed that IpaB levels in Δ*ansB* and wild type cells are comparable (Figure S1.B), which suggests that there may be an IpaB-independent mechanism involved in *S. flexneri* adhesion to host cells.

DIGE analysis was performed to identify changes in the *S. flexneri* proteome in response to mutating *ansB*. Comparative analysis of proteomes of the *ans*B mutant and wild type *S. flexneri* strains showed that this mutation exerts pleiotropic effects on the expression of several *S. flexneri* proteins (Table S1), including proteins involved in stress responses (DnaK and GroEL), translation (RplL), metabolism (Udp and Mdh) and an outer membrane protein (OmpA). OmpA is a prominent outer membrane protein found in Gram negative bacteria, required to maintain the structural integrity of cellular outer membranes [35], [36] and the role of this protein in Gram negative bacterial pathogenesis has been extensively studied [37]–[39]. Furthermore, studies in *E. coli* [40] and enterohemorrhagic *E. coli* (EHEC) [41], [42] have found that OmpA plays a key role in the initial stages of bacterial adhesion and invasion. In this study we show that AnsB is required for the adherence of *S. flexneri*, and *ansB* mutant cells up-regulate the expression of OmpA, therefore the effect of overexpressing OmpA in *S. flexneri* was investigated.

*ansB* mutants showed an up-regulation of OmpA, it is generally believed that the overexpression of membrane proteins affects the integrity of cell membranes and cell viability [43]. Therefore the overexpression of OmpA would affect cellular protein homeostasis, which in turn would have an effect on the overall integrity of the cellular envelope. Therefore to determine the effects of elevated OmpA levels on bacterial adherence, we overexpressed OmpA in the wild type strain and found that the overexpression of this outer membrane protein decreased both bacterial adherence to BHK cells and accumulation in *C. elegans* (Figure 7.A and B). These results suggest that the up-regulation of OmpA in *ansB* mutant cells could disrupt the bacterial cell envelope integrity and therefore may be one of the factors that contribute to the decreased adherence of these mutant cells.

qRT-PCR analysis of *ompA* expression in the *ansB* complement showed that complementation of the *ansB* mutation resulted in decreased *ompA* expression. This result suggests that the presence of *ansB* directly or indirectly affects *ompA* expression. The expression of OmpA has been known to be environmentally responsive. In *E. coli*, OmpA levels are elevated in response to nitrogen shortage, to facilitate an increased uptake of peptides to alleviate the nitrogen shortage [44], [45]. One of the identified functions of AnsB is to provide nitrogen and carbon sources from the hydrolysis of exogenous asparagine [46]. Nitrogen depletion increases the expression of *ansB* in yeast [47], [48], suggesting that AnsB plays a role in nitrogen metabolism. *ansB* mutant cells would thus have lower nitrogen levels compared with wild type cells; therefore we propose that the expression of OmpA in these mutant cells may be increased to alleviate this nitrogen shortage.

The exact mechanism involved in the AnsB-mediated regulation of OmpA remains unclear at this stage nonetheless our finding provided clear evidence suggesting that the presence of AnsB is required for the adherence of *S. flexneri* and for regulating OmpA levels.

## Conclusions

Here we have shown that the periplasmic enzyme, AnsB, is required for the virulence of *S. flexneri*. *in vitro* cell culture assays indicate that this enzyme is essential for bacterial adherence. Differential in-gel electrophoresis showed that *ansB* mutants exert pleiotropic effects on the expression of a number of *S. flexneri* genes, including a prominent bacterial outer membrane protein, OmpA that is known to be required for adherence to host cells. We also show that the up-regulation of OmpA, could be one of the factors responsible for the defective adherence of *ansB* mutant cells. This is the first report in *S. flexneri* where the function of AnsB has been found to extend beyond its canonical metabolic role. The requirement of AnsB for the virulence of *S. flexneri* makes this gene an attractive candidate for designing new therapeutic measures and preventive approaches to curb the spread of shigellosis.

## Supporting Information

Figure S1 ***ansB*** **mutation does not affect the expression and secretion of invasion protein antigens-IpaB and IpaD.** Secreted proteins were isolated from the *S. flexneri* serotype 3b parental strain (SFL1520), *ΔansB* (SFL2283) and a *S. flexneri* strain cured of the virulence plasmid (SFL1223) [20] used as a negative control. Proteins were separated using SDS-PAGE, following which they were electroblotted onto PVDF membranes and probed with anti-IpaB (**A**) and anti-IpaD (**B**) primary antibodies. The levels of secreted IpaB (∼62 kDa) and IpaD (∼34 kDa) produced by all strains was visualized using a chemiluminescence reader. No differences were observed in the secretory levels of both IpaB and IpaD between the wild type and *ansB* mutant strains.
(TIFF)

Figure S2 **AnsB activity is not required for** ***S. flexneri*** **growth** ***in vitro*** **.** The growth curves of wild type *S. flexneri* serotype 3b (SFL1520) (black) and Δ*ansB* strain (SFL2283) (grey), plotted as optical density readings at 600 nm (OD_600_) (y-axis) versus time (x-axis). **A**: Growth in Luria-Bertani (LB) broth at 37°C (bold lines) and at 30°C (dashed lines). **B**: Growth in minimal essential salts media (MM) at 37°C (bold lines) and at 30°C (dashed lines). No significant difference in the growth patterns of SFL1520 and SFL2283 were detected under all four conditions examined (*p*>0.05, unpaired t-test). Error bars represent standard error of means obtained from three independent biological repeats.
(TIFF)

Table S1 **Differentially expressed spots in the** ***ansB*** **mutant.**
(DOCX)
