# Supplementary figures and images for: The Periplasmic Enzyme, AnsB, of Shigella flexneri Modulates Bacterial Adherence to Host Epithelial Cells
Source: PLoS One. 2014 Apr 24;9(4):e94954. doi: 10.1371/journal.pone.0094954 (PMC3998974; doi:10.1371/journal.pone.0094954)

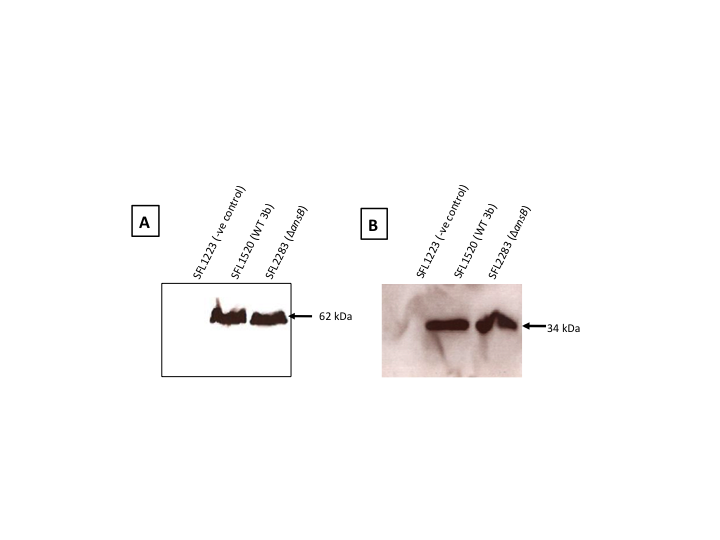

Supplement: Figure S1 — ansB mutation does not affect the expression and secretion of invasion protein antigens-IpaB and IpaD. Secreted proteins were isolated from the S. flexneri serotype 3b parental strain (SFL1520), ΔansB (SFL2283) and a S. flexneri strain cured of the virulence plasmid (SFL1223) [20] used as a negative control. Proteins were separated using SDS-PAGE, following which they were electroblotted onto PVDF membranes and probed with anti-IpaB (A) and anti-IpaD (B) primary antibodies. The levels of secreted IpaB (∼62 kDa) and IpaD (∼34 kDa) produced by all strains was visualized using a chemiluminescence reader. No differences were observed in the secretory levels of both IpaB and IpaD between the wild type and ansB mutant strains. (TIFF) [file pone.0094954.s001.tif]

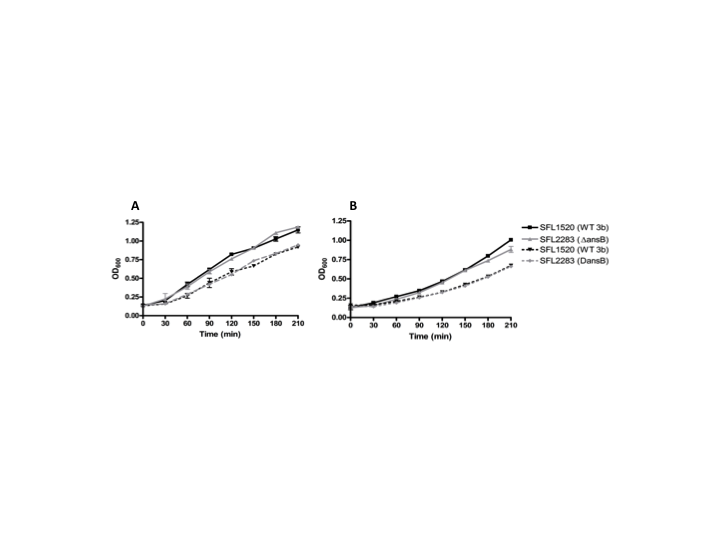

Supplement: Figure S2 — AnsB activity is not required for S. flexneri growth in vitro . The growth curves of wild type S. flexneri serotype 3b (SFL1520) (black) and ΔansB strain (SFL2283) (grey), plotted as optical density readings at 600 nm (OD600) (y-axis) versus time (x-axis). A: Growth in Luria-Bertani (LB) broth at 37°C (bold lines) and at 30°C (dashed lines). B: Growth in minimal essential salts media (MM) at 37°C (bold lines) and at 30°C (dashed lines). No significant difference in the growth patterns of SFL1520 and SFL2283 were detected under all four conditions examined (p>0.05, unpaired t-test). Error bars represent standard error of means obtained from three independent biological repeats. (TIFF) [file pone.0094954.s002.tif]
