# Supplementary material for: The Periplasmic Enzyme, AnsB, of Shigella flexneri Modulates Bacterial Adherence to Host Epithelial Cells
Source: PLoS One. 2014 Apr 24;9(4):e94954. doi: 10.1371/journal.pone.0094954 (PMC3998974; doi:10.1371/journal.pone.0094954)
Supplement: Table S1 — Differentially expressed spots in the ansB mutant. (DOCX) [file pone.0094954.s003.docx]

**Table S1: Differentially expressed spots in the *ansB* mutant**

| **Sopt No.** | **Gene name** | ***Differential expression** | **Accession number (UniProt)** | **Description** | **Predicted pI/Mwt (kDa)** | **Observed pI/Mwt (kDa)** | **Coverage (%)** | **n (peptides matched)** | **Score** | ***p* value (One way ANOVA)** |
| --- | --- | --- | --- | --- | --- | --- | --- | --- | --- | --- |
| 1 | rplL | 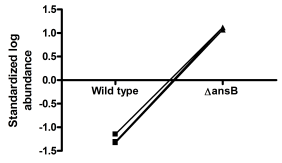 | Q0SY14 | ribosomal protein L7/L12 | 4.6/12.3 | 4.7/10.0 | 19 | 2 | 36 | 0.014 |
| 2 | udp | 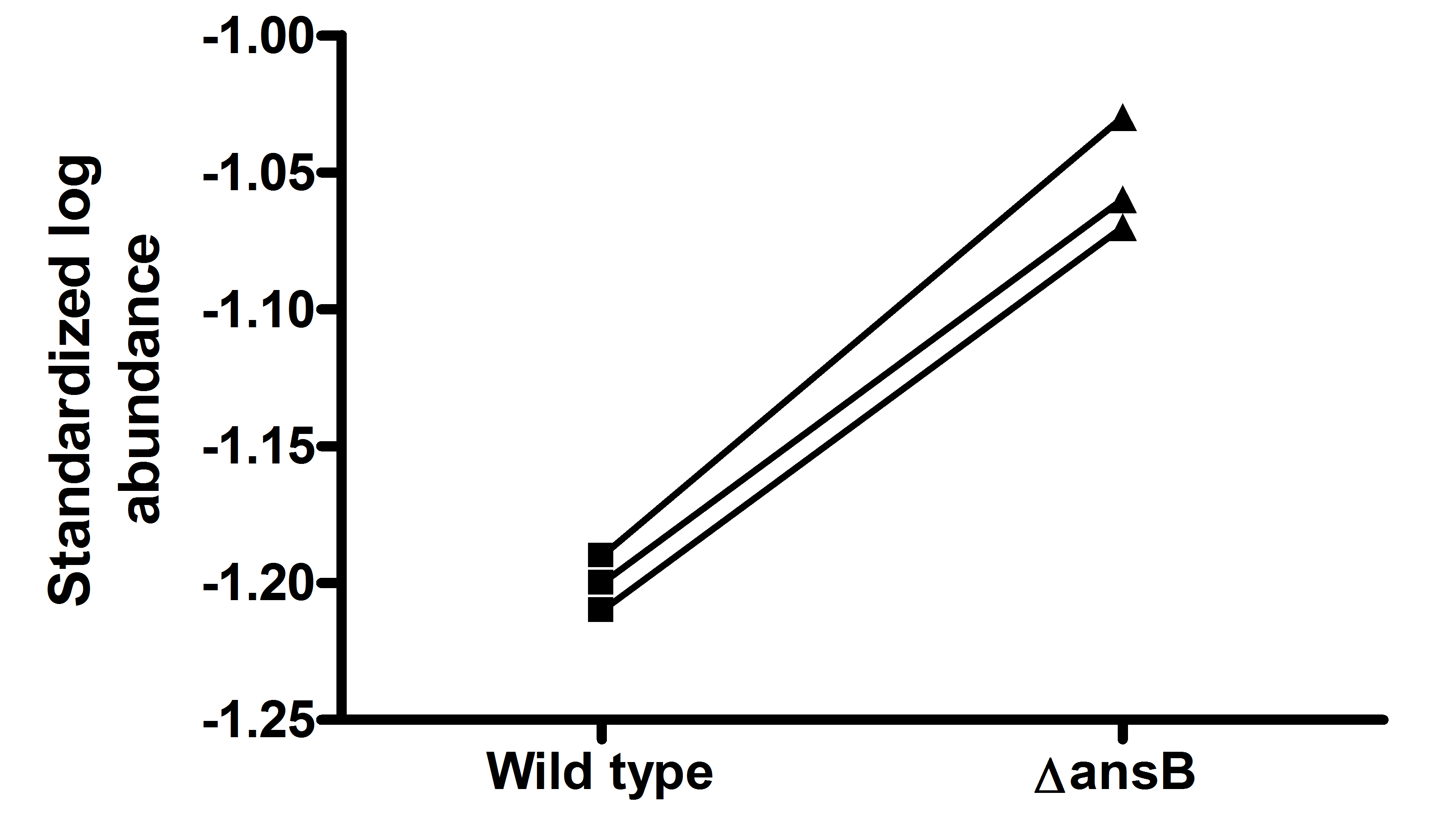 | F5MTS6 | Uridine phosphorylase | 5.81/27.3 | 5.8/28.0 | 32 | 4 | 41 | 2.7e-005 |
| 3 | mdh | 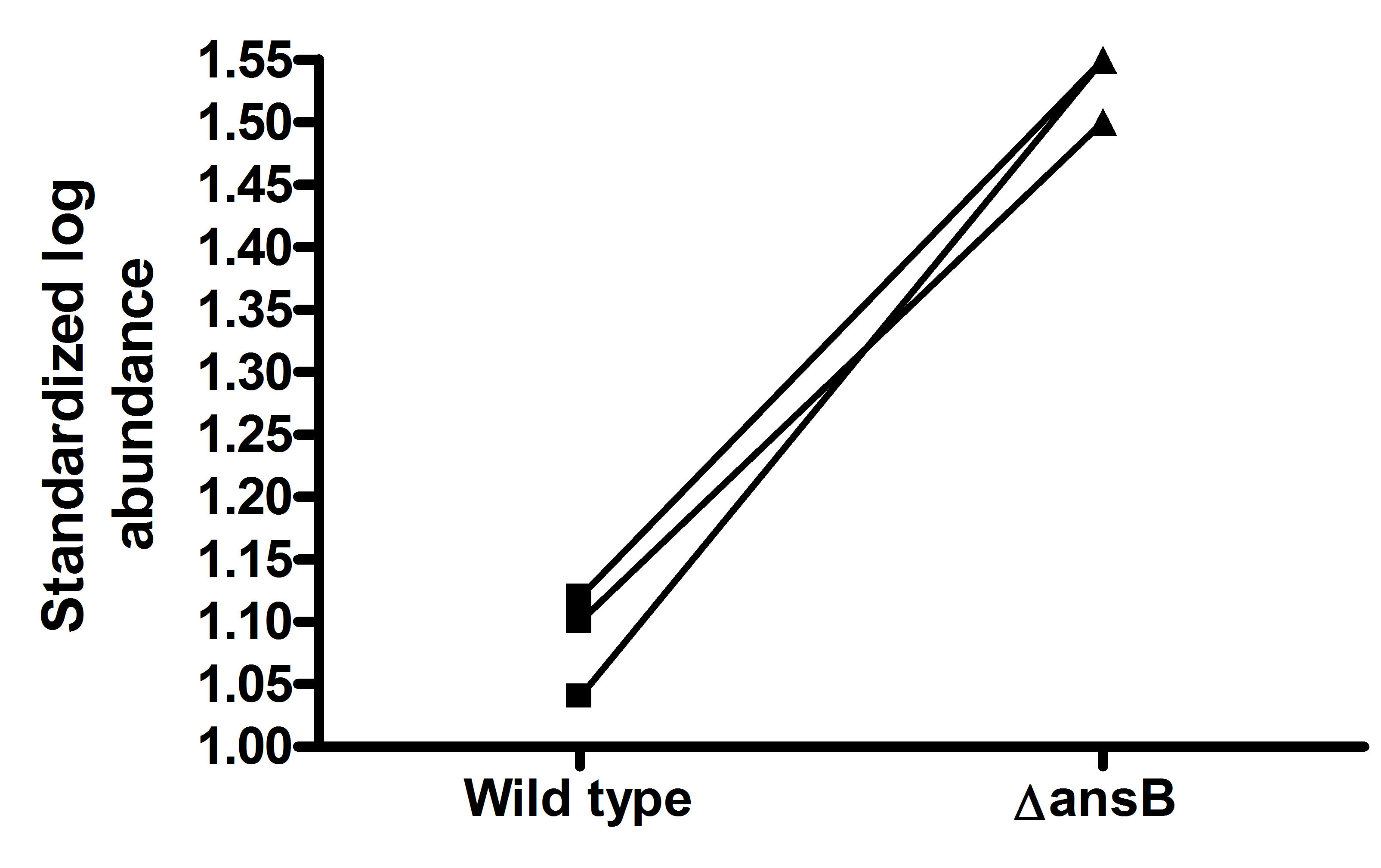 | F5NLQ6 | Malate dehydrogenase | 5.61/32.5 | 5.5/39.0 | 44 | 5 | 123 | 0.0041 |
| 4 | ompA | 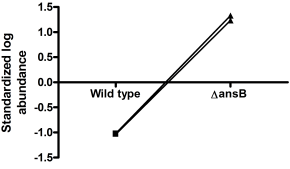 | Q0T678 | ompA Outer membrane protein 3a | 5.87/37.4 | 4.7/39.00 | 23 | 7 | 43 | 0.02 |
| 5 | groEL | 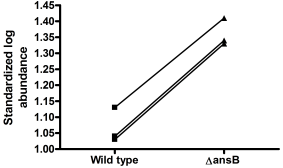 | Q0SXD6 | 60 kDa chaperonin | 4.85/47.46 | 4.8/67.00 | 29 | 11 | 130 | 0.047 |
| 6 | dnaK | 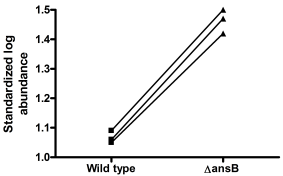 | I6CN04 | Chaperone protein DnaK | 4.83/69.13 | 4.8/6.9 | 31 | 13 | 175 | 0.00046 |

* Graphs depicting the differences in standardized log abundance of identified spots in wild type and *ansB* strains across three biological replicates calculated using the DeCyder version 5 software package.
